# IgA Vasculitis (Henoch–Schönlein Purpura): An Update on Treatment

**DOI:** 10.3390/jcm13216621

**Published:** 2024-11-04

**Authors:** Santos Castañeda, Patricia Quiroga-Colina, Paz Floranes, Miren Uriarte-Ecenarro, Cristina Valero-Martínez, Esther F. Vicente-Rabaneda, Miguel A. González-Gay

**Affiliations:** 1 Rheumatology Division, H. Universitario La Princesa, IIS-Princesa, 28006 Madrid, Spain; pquiroga@alumni.unav.es (P.Q.-C.); paz.floranes@salud.madrid.org (P.F.); miren.uriarte@salud.madrid.org (M.U.-E.); cvalero@salud.madrid.org (C.V.-M.); evicenter.hlpr@salud.madrid.org (E.F.V.-R.); 2 Department of Medicine, Universidad Autónoma de Madrid (UAM), 28049 Madrid, Spain; 3 Department of Medicine and Psychiatry, School of Medicine, University of Cantabria, 39011 Santander, Spain; 4 Rheumatology Division, IIS-Fundación Jiménez Díaz, 28040 Madrid, Spain; 5 Cardiovascular Pathophysiology and Genomics Research Unit, School of Physiology, Faculty of Health Sciences, University of the Witwatersrand, Johannesburg 2000, South Africa

**Keywords:** Henoch–Schönlein purpura, IgA vasculitis, IgA vasculitis nephritis, glucocorticoids, cyclosporine A, tacrolimus, mycophenolate mofetil, cyclophosphamide, rituximab, plasma exchange, immunoglobulins, experimental therapies

## Abstract

**Objective:** IgA vasculitis (IgAV), previously named as Henoch–Schönlein purpura, is the most frequent systemic vasculitis in children. In adults, IgAV is less common although it is associated with more severe disease. In fact, the frequency of glomerulonephritis (referred to as IgAV nephritis) in adults is higher than in children and tends to present more severely, with around 10–30% of those affected eventually progressing to end-stage renal disease. In this review, we describe the pathophysiology, main clinical features, diagnosis of the disease, and latest clinical data regarding IgAV therapy. **Methods:** A narrative literature review, primarily based on articles published in PubMed, was conducted. In addition to discussing the main aspects of glucocorticoids and conventional disease-modifying drugs used in the management of IgAV, this review focuses on the latest information reported regarding biologics and potential future therapies. **Results:** Glucocorticoids are the first-line therapy for IgAV, especially in adults with severe manifestations. Colchicine, dapsone, and methotrexate can be useful for controlling minor manifestations. Several immunomodulatory agents, such as cyclosporine A, tacrolimus, and mycophenolate mofetil, have shown favorable results as glucocorticoid-sparing agents. Leflunomide has shown promising results but requires further study. The use of rituximab has demonstrated efficacy in reducing relapse frequency, lowering the cumulative glucocorticoid burden, and achieving long-term remission of the disease in children and adults with IgAV. Immunoglobulins and plasma exchange therapy can also be useful in difficult and life-threatening situations. Other potential therapies with encouraging results include TRF-budesonide, B-cell-directed therapy, B-cell-depleting agents, sodium–glucose cotransporter-2 inhibitors, endothelin receptor antagonists, and complement pathway inhibitors. **Conclusions:** Glucocorticoids are the first-line therapy for IgAV, especially in adults with severe manifestations. The role of various immunomodulatory therapies, such as calcineurin inhibitors and mycophenolate mofetil, remains promising, while rituximab reduces the long-term side effects of glucocorticoids and can help achieve disease remission. Other potential therapies with encouraging results require further research.

## 1. Introduction

Henoch–Schönlein purpura (HSP) is a systemic small-vessel vasculitis whose main anatomopathological feature is the deposition of IgA1-dominant immune complexes (ICs) in the affected tissues, and its nomenclature has been recently changed to immunoglobulin A vasculitis (IgAV) in the latest consensual classification of vasculitis [1]. The classic clinical tetrad of IgAV include palpable purpura, gastrointestinal (GI) manifestations (such as abdominal pain or bleeding), arthralgia or arthritis, and glomerulonephritis [2,3,4,5]. An annual incidence of IgAV in children of around 3 to 27 cases per 100,000 cases has been reported, representing the most frequent vasculitis in childhood [6,7].

In Northwestern Spain, an epidemiological study conducted over 20 years found a higher prevalence of IgAV in children during fall and winter, and reported an annual incidence rate of 10.45 per 100,000 cases in children under 14 years of age [7]. In children, IgAV typically affects children between 4 and 7 years old, with a mean age of 6 years, and the course is generally benign, requiring only supportive treatment, unlike adults, in whom the disease tends to have a more severe presentation due to frequent GI or renal involvement, which worsens the prognosis. Additionally, the frequency of IgAV in adults is about 10 times lower than in children, with an annual incidence of 0.1 to 14 per 100,000 people [4,8,9]. IgAV in adulthood is more frequent in middle-aged people (male/female ratio = 1.5), although it can occur from 20 to 80 years of age [10].

The deposition of IgA1-dominant ICs in the small vessels of the main affected organs (skin, kidney and GI tract) is key in the pathogenesis of IgAV. Elevated serum IgA1 levels in patients may result from increased production or impaired clearance. Some evidence points to genetic susceptibility and specific antigen interactions in the mucosa as potential triggers. Additionally, aberrant glycosylation of IgA1 has been described as a predisposing factor for the IC formation, since it has been associated with galactose-deficient IgA1 (Gd-IgA1). On the other hand, the clinical and histological presentation of IgAV nephritis (IgAVN) can be similar to IgA nephropathy (IgAN), and some experts now consider these two conditions part of the named “IgA spectrum disease”.

## 2. Pathophysiology of IgA Vasculitis

The pathophysiology of IgAV is multifactorial, and involves complex interactions between the immune system, genetic predisposition, and environmental triggers. A hallmark of IgAV is the deposition of IgA1-dominant ICs in small vessels, which causes inflammation in the skin, kidneys, joints, and GI tract. Although IgAV shares certain pathological mechanisms with IgA nephropathy, the two conditions differ in their clinical presentation and outcomes.

There are several mechanisms that are implicated in the pathogenesis of the disease. One of them is an aberrant glycosylation of IgA1. In this regard, IgA1, which is one of the subclasses of IgA, is glycosylated at its hinge region through a process called O-glycosylation. In IgAV, defective glycosylation results in the formation of Gd-IgA1, which predisposes individuals to the production and deposition of pathogenic IC [11,12,13]. Gd-IgA1, produced primarily in response to mucosal infections, often following an upper respiratory infection, is thought to be a central player in IgAV pathogenesis. The increased production of Gd-IgA1 can take place in mucosal-associated lymphoid tissues (MALTs), like the tonsils or Peyer’s patches, and its defective clearance by the liver can lead to the deposits in the vessel walls and renal mesangium, triggering inflammation [14,15].

Another key point in the pathogenesis of IgAV is the formation of Gd-IgA1 IC. Gd-IgA1 interaction with autoantibodies (either IgA1 or IgG antibodies) leads to the formation of circulating IC. These Gd-IgA1 ICs then deposit in small vessel walls of the skin, kidneys, or GI tract, causing localized inflammation. The disease is not caused exclusively by the presence of elevated levels of circulating Gd-IgA1, which emphasizes the importance of IC formation and deposition in the pathogenesis of the disease [14,16,17].

Activation of the alternative and lectin pathways of the complement system by the Gd-IgA1 ICs also plays a relevant role [18]. Elevated deposits of complement components such as C3a, C5a, C4, and the membrane attack complex (C5b-9) are found in IgAV patients, and their presence correlates with disease severity, especially in cases involving renal complications [19]. Activation of complement pathways leads to cytokine production and recruitment of inflammatory cells, further amplifying tissue damage [14].

The activation of T-cells and B-cells is also relevant. With respect to this, mucosal infections can induce the production of IgA through both T-cell dependent and independent mechanisms. Class-switching of B-cells to produce IgA is promoted by dendritic cells that secrete cytokines such as IL-6, IL-10, as well as B-cell activation factor (BAFF) and a proliferation-inducing ligand (APRIL). T-cell activation in response to antigen presentation via HLA molecules also contributes to autoimmunity, and may play a role in IgAV susceptibility.

As occurs in other autoimmune diseases, environmental triggers may promote the development of the IgAV. Mucosal infections often precede IgAV onset, and several pathogens have been implicated in triggering the disease, such as viruses (Hepatitis B or parvovirus) or bacteria, among which Streptococcus, Staphylococcus or Helicobacter pylori stand out [20]. Recent reports also suggest an association between COVID-19 and IgAV, as well as some vaccines, which may either exacerbate or initiate the disease in susceptible individuals [21,22,23,24,25]. Infectious agents may express molecular mimics of vessel wall antigens, leading to the production of cross-reactive antibodies, or they may modify the glycosylation of IgA1, facilitating Gd-IgA1 production [14].

Like IgAV, IgA nephropathy is often preceded by an upper respiratory tract infection and usually presents with hematuria, hypertension, and other unimpressive clinical manifestations. Underlying pathological mechanisms involving abnormal O-glycosylation, IgA1 IC creation, and glomerular deposition, as well as involvement of the complement cascade and T-cell independent mechanisms appear to be shared between IgA nephropathy and IgAVN. Therefore, drugs which are being proved in IgA nephropathy might also have benefit in IgAVN.

Finally, genetic susceptibility has been described in patients with IgAV. Whereas no specific mutations have been definitively linked to IgAV, there is evidence of genetic susceptibility. Geographic and ethnic differences in IgAV prevalence, as well as familial aggregation, point toward genetic predisposition [26]. The human leukocyte antigen (HLA) region has been identified as the main genetic factor linked to the pathogenesis of IgAV [14]. Both HLA class I and class II alleles seem to be involved in genetic predisposition of this vasculitis, although a stronger association with HLA-DRB1 alleles has been reported. MHC molecules appear to play a role in antigen presentation and immune regulation. This genetic susceptibility may influence the immune system’s response to environmental triggers, increasing the risk of IgAV in certain individuals [13].

In summary, the pathophysiology of IgAV involves a combination of genetic predisposition, environmental factors, in particular infections, and immune dysregulation, with Gd-IgA1 and ICs formation playing a central role. The deposition of these complexes in small vessels triggers a local inflammatory response, involving both complement activation and the recruitment of immune cells, leading to the characteristic clinical features of the disease.

## 3. Clinical Manifestations

The development of palpable purpura, arthralgia, gastrointestinal and renal involvement over days to weeks are considered the classic tetrad of IgAV manifestations. Purpura and arthralgia are usually the initial manifestations, although the disease can also begin with renal or GI tract involvement [27,28].

Cutaneous manifestations are present in nearly 100% of patients, with palpable purpura being the most common. It is typically symmetrical, and predominantly affects dependent areas such as the lower limbs, back, and buttocks. Up to one-third of adults may present with hemorrhagic or necrotic lesions. The lesions usually last for around 10 days.

Abdominal pain is observed in 50–75% of patients and results from vasculitic involvement of the GI tract [4,27,29,30,31,32,33]. The pain is typically diffuse, colicky, or anginal, sometimes periumbilical, and may be accompanied by nausea, vomiting, diarrhea, rectal bleeding, and melena. In some cases, it can mimic an acute abdomen. Thirty percent of patients experience intestinal bleeding, so it is recommended to perform a fecal occult blood test in those without obvious digestive bleeding to detect subclinical disease. Severe abdominal complications such as bowel infarction, perforation or intussusception require preferential or urgent surgery; otherwise, they can be fatal [4,27,29].

Joint involvement (arthralgia/arthritis) has been described in 60–80% of patients and is more common at disease onset in adults [27]. The most frequent presentation is arthralgia or a pattern of oligoarthritis, predominantly affecting the lower limbs, although the hands and feet can also be affected. Pain, swelling and functional impairment are the most characteristic joint manifestations [27,34].

Renal involvement in IgAV ranges from 40% to 85%. Nephropathy clinical spectrum includes conditions from simple microhematuria to nephritic syndrome, nephrotic syndrome, or renal failure [5,27]. In adults with IgAV, the frequency of glomerulonephritis (referred to as IgAV nephritis) is higher than in children and tends to present more severely, with around 10–30% of those affected eventually progressing to end-stage renal disease (ESRD).

Persistent melena or skin lesions lasting more than two months at disease onset have been considered to be risk factors for glomerulonephritis.

In this review, we have focused our attention on the pathogenesis, clinical presentation and diagnostic process of IgAV. The severity of the initial renal presentation determines the prognosis. A renal biopsy is recommended in cases of marked proteinuria, persistent active urinary sediment, or prolonged significant hematuria. The presence of crescents and glomerular sclerosis is also associated with a poor prognosis. Renal biopsy may reveal various histological patterns, with mesangial proliferation being the most typical lesion [2,5]. Mesangial IgA deposition seen on kidney biopsy in IgAVN is indistinguishable from that observed in IgA nephropathy, and the consensus amongst experts is to accept these two diseases as an extension of each other.

Gastrointestinal involvement, when severe, is what determines the severity and short-term prognosis, while the long-term prognosis of the disease is determined by renal manifestations, which lead to either chronic kidney disease (CKD) or ESRD in 10–30% of adult-onset IgAV [6].

Less common clinical manifestations of IgAV include neurological symptoms (such as headaches, seizures, ataxia, mono/polyneuropathy) and cerebral vasculitis. Non-specific central-nervous-system manifestations can pose a diagnostic challenge, as they include symptoms such as irritability, dizziness, behavioral changes or emotional instability, in addition to testicular bleeding, and interstitial lung disease or alveolar pulmonary hemorrhage [35].

Relapses are observed in almost a third of patients with IgAV [35,36,37], and generally occur soon after the first episode of vasculitis [37].

## 4. Diagnosis

Clinical manifestations and histopathological findings constitute the basis for the diagnosis of IgAV. Several IgAV classification criteria have been published, including the following: 1990 American College of Rheumatology (ACR) criteria [38], the European League Against Rheumatism (EULAR) criteria, the Paediatric Rheumatology International Trials Organization (PRINTO) criteria, and the 2010 European Paediatric Rheumatology Society (PRES) criteria [39]. The latter criteria, specifically aimed at the pediatric population, include the presence of IgA deposits as an independent criterion (Table 1), and have a 100% sensitivity and 87% specificity in this population [39]. Their sensitivity and specificity in the adult population with IgAV have also shown very favorable values (99.2% and 86%, respectively), which supports their use, regardless of age [40] (Table 1).

Currently, we cannot rely on diagnostic biomarkers for IgAV, as none of the proposed ones have proven clinically useful, although they could have some value in determining disease activity or prognosis [41,42,43]. Elevated serum levels of IgA, present in more than 50% of patients, have no diagnostic or prognostic value.

In children, the diagnosis is usually clinical and does not require histological confirmation, whereas in adults histological confirmation is recommended. Skin biopsy typically shows leukocytoclastic vasculitis, with IgA and complement (C3) deposits visible by direct immunofluorescence techniques.

The differential diagnosis, especially in the adult population, must be made mainly with hypersensitivity vasculitis, cryoglobulinemic vasculitis, microscopic polyangiitis and polyarteritis nodosa.

## 5. Treatment of IgA Vasculitis

The treatment of IgAV remains controversial. Treatment of mild skin, joint and GI manifestations is usually symptomatic, including general measures, adequate hydration and analgesics. However, the need to use immunosuppressive (IS) therapy is often determined by the presence of renal involvement. Nonsteroidal anti-inflammatory drugs (NSAIDs) can be beneficial in cases of arthralgia or arthritis in patients without renal or intestinal affection for short periods of time, as long as the patient does not have renal or GI involvement, in which case they should be avoided, due to the risk of deterioration of renal function and GI bleeding. Adequate care of skin wounds is essential in cases of necrosis and ulceration. The appearance of edema in the lower limbs may benefit from the use of compression stockings, and in the case of mild-to-moderate proteinuria or elevation of blood pressure, it is advisable to associate treatment with angiotensin-converting enzyme (ACE) inhibitors (ACEI) and/or angiotensin receptor blockers (ARB). When internal and/or severe organic complications appear, glucocorticoids (GCs) alone or combined with IS agents are recommended, although there is still no consensus on the efficacy of these treatment regimens [5,13,27]. Additionally, severe GI complications such as perforation or intussusception may require preferential surgery. It is important to emphasize that most of the available information comes from studies in the pediatric population, so the data from the adult population are the result of extrapolation. However, and although it is difficult to separate both scenarios, in this section we will place special emphasis on adult-onset IgAV, since this is where most of the important complications and sequelae can appear.

### 5.1. Glucocorticoid and Other Immunosuppressive and Immunomodulatory Agents

#### 5.1.1. Glucocorticoids

Glucocorticoids (GCs) are particularly useful in the acute and early stages of the disease, due to their anti-inflammatory efficacy and rapid onset of action. In a meta-analysis by Weiss et al. comprising 12 retrospective studies and 3 randomized clinical trials, the use of GCs resulted in earlier resolution of abdominal pain, as well as a reduction in the risk of relapses, chronic kidney disease or complications requiring surgery [44]. Nevertheless, there is currently no consensus on whether to use GCs in the absence of renal involvement, and much less in children in whom the course of the disease is usually benign. Dudley et al. [45] published a double-blind, randomized, placebo-controlled trial evaluating the effect of GCs in 352 children with recent-onset IgAV and minor or absent kidney disease. The authors concluded that the administration of prednisolone, 2 mg/kg/day during the first 2 weeks and a dose of 1 mg/kg/day the following week, did not show a significant reduction in the risk of proteinuria versus placebo at 12 months of follow-up [45]. In contrast, another study also conducted in a pediatric population found a faster resolution of hematuria and proteinuria with GCs than with placebo, with renal manifestations disappearing at 6 months in 61% and 34% of patients, respectively, despite the fact that GCs did not prevent their appearance after 28 days of therapy. This effect was particularly evident in patients older than 6 years who had mild renal manifestations at the inclusion, suggesting the possible usefulness of GCs in mild cases to modify the course of renal involvement [46]. In keeping with this, Niaudet et al. conducted a prospective, uncontrolled study in IgAVN that showed a complete recovery of renal failure and/or nephrotic syndrome at the last follow-up (mean, 5.6 years) in 27 of 38 children after administration of 3 boluses of methylprednisolone (MP) followed by more than 3 months of treatment with oral prednisone [47].

In a 2015 Cochrane review including five randomized controlled trials, there was no clear evidence of benefit of GCs for renal involvement in patients with IgAV [48]. In another study comparing MP and cyclosporine A (CsA) in 24 children with IgAV nephritis with nephrotic-range proteinuria or crescent-shaped lesions on renal biopsies, CsA was associated with earlier resolution of proteinuria and a higher response rate in biopsy results at two-year follow-up [49].

With respect to adult-onset IgAV nephritis, we must highlight the scarcity of information that compares the usefulness of supportive therapy versus GCs from randomized studies. Indeed, much of the available data come from IgA nephropathy trials. Pozzi et al. [50] compared supportive care in IgA nephritis versus GCs in a controlled randomized trial including patients with proteinuria (1–3.5 g/day) and renal failure (serum creatinine < 1.5 mg/dL). The administration of 3 consecutive boluses of 1g MP (1, 3, and 5 months) followed by 6 months of oral prednisone (0.5 mg/kg/48 h) protected against deterioration in renal function without significant toxicity [50]. An extension of this trial showed that GCs were associated with a significantly improved renal survival and 24 h proteinuria at ten years of follow-up, compared with supportive care [51]. Accordingly, in IgAV nephritis with moderate/severe proteinuria and a GFR > 50 mL/min, GCs are the recommended therapy by the KDIGO guidelines, while for IgA-crescentic nephritis with nephrotic syndrome and/or impaired renal function, IS such as cyclophosphamide (CYC) is reserved [52]. IgAV forms with severe skin involvement, such as hemorrhagic vesicles, bullae, ulcers and/or necrosis, unlike the classic forms, require GC therapy more frequently because they have a more torpid evolution. In a retrospective study collecting data from 12 tertiary medical centers, pediatric patients with (*n* = 64; median age of 8.08 years) and without (*n* = 596; median age of 6.33 years) severe IgAV/HSP skin changes were compared [53]. It showed a significant increase in the risk of developing nephritis (OR = 3.1 [95% CI: 1.04–9.21]; *p* = 0.042) and GI complications such as hematochezia, massive bleeding and/or intussusception (OR = 3.65 [95% CI: 1.08–12.37]; *p* = 0.038) in patients with severe skin involvement in the multivariate analysis [53]. The same may apply to adults with IgAV and purpura with severe skin manifestations such as blisters, ulcerations, necrosis and hemorrhagic lesions, for which GCs might be equally advisable.

#### 5.1.2. Azathioprine

The evidence in favor of the use of azathioprine is limited to an old observational study in 20 patients with severe IgAVN of pediatric onset, in which, after a follow-up of 4.8 years, clinical remission was achieved in 60% of the patients treated with azathioprine in combination with GCs. In contrast, current data do not support its efficacy, and even suggest potential toxicity in IgA nephropathy [54].

The long-term use of azathioprine in combination with low-dose prednisolone did not show a benefit in clinical evolution compared to untreated controls in a retrospective study (*n* = 74 patients) with a 10-year follow-up. Its association with GCs also had no impact on renal survival in a recent prospective study that included 207 patients with IgA nephropathy [54]. Therefore, treatment of IgAN with azathioprine is not currently recommended [55].

#### 5.1.3. Mycophenolate Mofetil

The mechanism of action of mycophenolate mofetil (MMF) has been linked to the inhibition of type I and II inosine monophosphate dehydrogenase, which inhibits the proliferation of B and T lymphocytes reversibly, since the de novo synthesis of DNA and guanosine nucleotide is prevented [56].

Evidence has been published on the effectiveness of MMF in both adult and pediatric populations for the treatment of IgAV nephritis and IgA nephropathy. In children, two case series (*n* = 6 in 2010; *n* = 12 in 2012) showed that MMF induced remission and prevented flares in GC-refractory or GC-dependent IgAV nephritis [57,58]. In line with this, in a retrospective study conducted by Ren et al. [59] in 53 adult patients with biopsy-proven IgAV nephritis and proteinuria (>2 g/day), the use of MMF in combination with low-dose prednisone was associated with lower rates of relapse than with high-dose prednisone, although no differences in remission rates were found [59]. Additionally, in a retrospective study including 95 adult patients with IgAV who met the 2010 EULAR/PRINTO/PRES classification criteria and had persistent proteinuria, despite treatment with renin–angiotensin–aldosterone system (RAAS) inhibitors, MMF combined with low-dose glucocorticoids (0.4–0.5 mg/kg/day) was shown to be more effective than RAAS inhibition alone in reducing proteinuria and achieving remission after one year of treatment, with fewer adverse effects than high-dose glucocorticoid (0.8–1.0 mg/kg/day) monotherapy [60].

Along the same lines, the higher remission rates achieved with MMF versus controls has also been suggested by a meta-analysis that included eight studies of IgA nephropathy [61] and a Chinese randomized clinical trial that included 170 adults with progressive IgA nephritis, in which the association of MMF with supportive treatment was associated with a decreased risk of progression of renal involvement [62]. Finally, MMF in combination with GCs has shown a better efficacy and safety profile than CYC in combination with GCs, in terms of higher rates of complete remission, with fewer adverse events in childhood IgAVN at 6 and 12 months of follow-up, in a recent meta-analysis by Wang et al. that included 675 patients from 10 studies [63]. Taking all this into consideration, we can conclude that MMF may be considered a GC-sparing agent, although long-term studies are needed to clarify its potential in the treatment of IgAV.

#### 5.1.4. Calcineurin Inhibitors

Calcineurin inhibitors (CNIs), more specifically cyclosporine A (CsA) and tacrolimus (TAC), are characterized by the inhibition of T lymphocyte activation. CsA has been tested for the treatment of adult IgAV/IgAV nephritis in a few studies of limited sample size and quality. A significant reduction in proteinuria related to IgA nephropathy induced by CsA has been reported in a prospective single-blind randomized study [64] and a potential renal protection of CNIs in IgA nephritis was suggested by a recent meta-analysis, but with the limitation of being associated with a higher risk of adverse effects [65]. At present, there is no robust evidence supporting their use in this context.

In children, the efficacy of CsA treatment for renal involvement of IgAV has been reported in some isolated cases [66,67] and in a randomized study, in which Jauhola et al. included 24 patients with severe IgAVN (nephrotic-range proteinuria and/or crescentic glomerulonephritis) and found that the group treated with CsA for one year presented an earlier remission and a lower relapse rate than the group treated with prednisone (3 boluses of MP + 4 months of oral prednisone), with a good safety profile [49]. Partial or complete remission of severe adult-onset IgAVN, with nephrotic-range proteinuria, after treatment with CsA combined with GCs has also been described in a small case series, while renal function remained stable at the five-year follow-up [68].

Regarding TAC, in the short term it seems to significantly decrease proteinuria in the pediatric population with IgAV [69]. Its off-label use has also shown therapeutic potential in children with refractory IgAVN, with persistent proteinuria despite high-dose CYC or MMF, in a retrospective study conducted by Gan et al. [70]. In this context, 37.5% and 31.2% of patients achieved complete and partial remission of proteinuria, respectively, while five patients (31.2%) did not achieve remission. According to these results, TAC may be an effective treatment option for children with refractory IgAVN, particularly in achieving proteinuria remission. However, further research to establish long-term efficacy and safety is required [70].

#### 5.1.5. Cyclophosphamide

Cyclophosphamide (CYC) is an alkylating agent used in the treatment of some glomerular diseases that inhibits DNA synthesis and crosslinks DNA strands to prevent cell division, leading to cell death. In rapidly progressive IgA nephritis, due to the high risk of ESKD and the absence of alternative rescue therapies, the KDIGO guidelines recommend the use of CYC in combination with GCs [71], although CYC did not demonstrate complete remission or prevention of ESKD in a Cochrane review [72].

With respect to IgAV nephritis, there is also no strong evidence on its efficacy. In the only randomized study in pediatric patients with histologically confirmed severe IgAVN, CYC (90 mg/m^2^/day) did not show significantly superior efficacy to supportive care in terms of partial or complete renal remission, or in the frequency of progression to ESKD after a seven-year follow-up [73]. In adults, Pillebout et al. also failed to demonstrate the superiority of CYC treatment combined with high-dose prednisone versus high-dose prednisone alone in patients with severe biopsy-proven IgAV (proliferative glomerulonephritis and/or severe GI disease) in the only prospective multicenter randomized trial available [74]. Efficacy and safety outcomes did not show significant differences between groups at either 6 months or 12 months, although survival was numerically higher in the CYC-treated group [74]. Similarly, combined use of CYC and GCs also showed no significant differences in partial or complete responses at 12 months compared with GCs monotherapy in the largest retrospective series of patients with adult-onset IgAV reported by Audemard-Verger et al., questioning the efficacy of CYC in this population [35].

#### 5.1.6. Other Synthetic Disease-Modifying Anti-Rheumatic Drugs (DMARDs): Colchicine, Dapsone, Hydroxychloroquine, Methotrexate, and Leflunomide

Colchicine is an alkaloid drug derived from the plants of the *genus Colchicum*, which are native to Europe and Eastern Asia. It possesses properties that inhibit several steps in the inflammatory process [75]. The ability of colchicine to inhibit the migration of polymorphonuclear cells to areas of inflammation is considered the mechanism responsible for its efficacy in cutaneous leukocytoclastic vasculitis [76], and the basis for its successful application to the treatment of cutaneous manifestations of IgAV in children and adults, by alleviating symptoms and improving quality of life [77,78]. Nevertheless, there is currently no solid justification to support its use.

Mechanisms that have been postulated to be responsible for the immunomodulatory properties of dapsone include inhibition of neutrophil free-radical production, synthesis of prostaglandin D2 and IgG and IgA antibodies, and interactions between neutrophils and IgA [79]. Rapid improvement of the rash associated with IgAV purpura has been described with dapsone (1–2 mg/kg/day) in adults and young patients [79,80]. However, the fact that it recurs rapidly after withdrawal suggests that dapsone does not completely suppress cutaneous vasculitis. In children, dapsone has been shown to be effective in treating the GI symptoms of IgAV, although there is no evidence of renal efficacy [81]. Adverse events include hemolysis and methemoglobinemia, which require special caution in patients with glucose-6-phosphate dehydrogenase deficiency.

Hydroxychloroquine (HCQ) is an antimalarial drug whose immunomodulatory properties with respect to IgAVN are based on its ability to inhibit both innate and adaptive immunity, stimulating the production of Gd-IgA1 [82]. In addition, it is used as a treatment for multiple autoimmune diseases such as rheumatoid arthritis (RA) and systemic lupus erythematosus (SLE). In a systematic review, HCQ has been shown to significantly reduce proteinuria in patients with IgA nephropathy, with a good safety profile, although without beneficial changes on the estimated glomerular filtration rate (eGFR) [82]. However, a reduction in proteinuria related to IgAVN has been reported in a Chinese retrospective case-control study [83].

Methotrexate is a synthetic immunomodulator that has been shown to be useful for the treatment of joint manifestations associated with IgAV, especially in cases of resistant or refractory arthritis. In the pediatric age, in the absence of renal involvement, MTX appears to be effective in sustaining remission, and it allows for the reduction in the use of steroids [84,85]. Regarding its role in adult-onset IgAV, we have no information in the literature.

Leflunomide (LEF) is another synthetic DMARD, widely used in many immune-mediated diseases. Although data on its role in IgAV are very preliminary and come from studies of low methodological quality, its addition to GCs has shown a beneficial effect on proteinuria in children [86] and on renal outcomes in adults [87]. We hope to have more clarifying data when the results of an ongoing randomized controlled trial (RCT) in patients with IgAV nephritis become available.

#### 5.1.7. Mizoribine

Mizoribine (MZB) is an imidazole nucleoside that reduces B- and T-cell proliferation and DNA synthesis through its inhibitory action on inosine monophosphate dehydrogenase and guanosine monophosphate synthetase [88] (Table 2). It also regulates the activation of heat shock protein (HSP)60, with the consequent potentiating effect on glucocorticoid receptors [89]. The expectations for MZB as a therapeutic agent in autoimmune diseases such as lupus nephritis are promising, based on its beneficial effect on nephrotic syndrome [90].

Currently, there are limited studies and case reports regarding the use of MZB in children and adults with IgAV [91,92].

The efficacy of MZB (150 mg/day) combined with a course of prednisolone was retrospectively evaluated by Mima and col. in five patients with adult-onset IgAV [92]. Remarkably, all patients achieved complete or partial remission of proteinuria and microscopic hematuria. No significant adverse effects were noted. Despite the small, uncontrolled nature of the study, the authors concluded the potential usefulness of this drug in this pathology [92].

### 5.2. Biological and Other Advanced Therapies

#### 5.2.1. Rituximab

Rituximab (RTX) binds to CD20+ lymphocytes causing a drastic decrease in B cells through complement- and antibody-dependent cellular cytotoxicity mechanisms [93]. Currently, RTX use is mainly restricted to refractory IgAVN, due to the scarce evidence on its efficacy regarding renal outcomes or Gd-IgA1 levels, which is supported mainly by isolated clinical reports and case series in both pediatric and adult patients [94,95,96]. Based on these cases, Maritati et al. conducted a multicenter observational study involving 22 adult patients with relapsing or refractory IgAV, or with contraindications and/or adverse events with respect to GCs or IS drugs [97]. RTX treatment showed a 91% remission rate at six months, with only one patient developing ESRD, and a 5-fold decrease in proteinuria. Furthermore, RTX appeared to be effective for relapse cases, even when used as monotherapy, and displayed a good safety and tolerability profile [97]. The data seem favorable, pending the availability of more robust evidence from randomized studies with adequate sample size.

In another study by Fenoglio et al. [98] including 12 adult-onset IgAV patients (mean age 45.1 years) with aggressive glomerulonephritis with biopsy-proven crescentic nephritis, RTX was administered to treat refractory disease or due to contraindications to conventional therapy. Remarkably, eleven patients (91.7%) achieved a clinical response at 6-month follow-up. In ten cases, the response was complete, while one patient needed an additional dose of RTX to achieve complete response six months later. During follow-up, two patients needed additional maintenance or induction doses of RTX due to relapse, with adequate response. The Birmingham Vasculitis Activity Score (BVAS) (*p* = 0.031) and 24 h proteinuria (*p* = 0.043) experienced a significant reduction. These results suggested that RTX could be a therapeutic alternative for refractory cases, but also could be used as first-line treatment [98].

At present, there are several studies in progress evaluating the efficacy of RTX in IgAV. The first one is a phase III randomized controlled trial comparing RTX in combination with GCs versus placebo, for newly diagnosed and/or refractory adult-onset disease. The second one is a phase II, modified crossover study examining the efficacy of infliximab, RTX, and tocilizumab in the treatment of refractory non-anti-neutrophil cytoplasmic antibody (ANCA)-associated vasculitis (AAV), including IgAV, in both adults and children (the ‘BIOVAS’ trial). Results of this trial are expected by the end of 2025, and could guide the treatment of refractory IgAV. Nevertheless, the absence of adequate responses observed in the trials evaluating RTX for IgA nephropathy must be highlighted, with the potential explanations being of a multiple nature [99].

Finally, other selective B cell-depleting agents such as ofatumumab, a fully humanized anti-CD20 monoclonal antibody, are being investigated. This drug, currently only approved for the treatment of multiple sclerosis, induced complete remission in a series of patients with severe IgAVN who experienced adverse events with RTX, suggesting a better tolerance of ofatumumab [100].

#### 5.2.2. Immunoglobulin Therapy

The use of immunoglobulins (IGs) may be effective in some cases of IgAV, due to its favorable effects at several levels of the immune system [101].

In a prospective study by Rostoker et al. conducted in 1994 [102], high-dose IG (2 g/kg monthly intravenous (IV) doses given for three months, followed by fortnightly intramuscular (IM) doses for six months) were administered to treat severe IgA nephropathy, either idiopathic or IgAVN, with poor prognosis indicators, obtaining very encouraging results. Nephrotic-range proteinuria decreased substantially, and the deterioration of the GFR slowed down, with good tolerance to therapy. Additionally, the histologic index of activity and the staining intensity of glomerular IgA and C3 deposits also diminished [102].

Based on these data, these authors later conducted a prospective open-label study including 14 patients with moderate IgA nephropathy (11 idiopathic cases and 3 IgAV) and mild-to-moderate proteinuria. Patients were treated with low doses of polyvalent IM immunoglobulins (IMIG) during 9 months. IMIG was well tolerated, with only one patient withdrawing from the trial. Most of the patients (12/13) experienced a favorable evolution. Albuminuria and the histological activity index, when available, were significantly reduced. Additionally, further reductions in IgA ICs, serum IgA and beta-2 microglobulin levels were observed, together with an elevation of serum IgG1 levels [103].

However, IG therapy has not been widely used since then, probably due to the high costs and limited availability; therefore, it is currently not well positioned in the therapeutic hierarchy of IgAV. However, it could be especially useful when active disease affects patients with concomitant infection. In this regard, the administration of 5 days of high-dose IVIG treatment to fourteen patients with vasculitis (one of them with IgAV) and simultaneous infection led to complete remission in seven patients (50%), partial remission in three (21%) and lack of response in four patients (29%) in an observational retrospective study. After a 24-month follow-up, the recurrence-free time in patients who had a favorable clinical response was close to 21 months, suggesting that IVIG may be a potential therapeutic alternative in situations where IS cannot be used [101].

#### 5.2.3. Plasma Exchange Therapy

Plasmapheresis or plasma exchange (PLEX) therapy is an invasive therapeutic procedure consisting in the removal of blood plasma, including some of its main deleterious components such as autoantibodies and circulating ICs. ANCA-associated vasculitis, rapidly progressive glomerulonephritis (RPGN) due to anti-glomerular basement membrane antibody disease and crescentic glomerulonephritis have shown favorable response to PLEX [104,105,106]. The pathogenetic involvement of circulating ICs in IgAVN is the rationale for the potential benefit of PLEX in severe renal impairment. The 2019 American Apheresis Society guidelines establish an individualized assessment of PLEX treatment in cases of IgA nephritis and IgAV with rapidly progressive disease, although with a weak level of recommendation due to the lack of prospective or randomized trials [107].

Three uncontrolled case series, which included a total of 32 children with severe IgAV nephritis/IgA nephropathy, have evaluated treatment with PLEX, either in monotherapy or in combination with IS. A very significant reduction in proteinuria and a dramatic improvement in renal function were observed, as well as remission of cutaneous and GI symptoms during treatment [108,109]. In another series including 11 adults with severe manifestations of IgAV, PLEX was used in combination with GC (IV MP pulses plus 6 months of oral prednisone), leading to fast reduction in proteinuria and BVAS, as well as to renal function improvement at 12 months of follow-up, with a median free-time of relapses of 6 years [110].

A recent systematic review has evaluated the efficacy and safety profile of PLEX for the treatment of RPGN (62.8% with IgA nephropathy and 37.2% with IgA/HSP) [106]. Most patients were men (69%), and the use of PLEX in combination with GCs and IS (61.6% CYC) was predominant, with a median of 3 to 18 sessions administered. In 29 of 38 patients with IgAVN treated with PLEX, remission was achieved (68.4% complete and 7.8% partial), while 23.6% (*n* = 9/38) developed ESKD. The authors concluded that adjunctive PLEX treatment with IS could be beneficial for RPGN patients, and that the better long-term results would be at the kidney level [106].

### 5.3. Potential Future Drugs for IgA Vasculitis Treatment

Recognizing suitable drug targets for this condition is really challenging, due to its predominance in childhood and its high probability of spontaneous remission. Therefore, potential new treatments to be used should be assessed based on risk stratification and meeting several requirements: cost-effectiveness, formulation appropriate for children and young people, and a good safety profile.

Regarding pathophysiology, IgA nephropathy and IgAV appear to share pathogenic mechanisms such as the synthesis of IgA1 IC, alterations in O-glycosylation, the deposition of ICs in the glomerulus or the involvement of B and T cells and the complement cascade. Currently, most studies are carried out in IgA nephropathy, a condition usually more severe than IgAV, but the possibilities of including patients with IgAV or IgAVN have been growing in recent years. A summary of the potential future therapies for IgAV is shown in Table 2 [111].

#### 5.3.1. TRF-Budesonide

The oral targeted-release formulation (TRF) of budesonide decreases Gd-IgA1 levels by targeting Peyer’s patches located within the distal ileum. In adult-onset IgA nephropathy, proteinuria and the risk of developing ESKD decreased significantly with this therapy, as reported by a large phase-IIb trial.

The efficacy and safety profile of TRF-budesonide has also been investigated in another randomized controlled phase-3 clinical trial including patients with primary IgA nephritis who are at risk of progressing to kidney failure. Remarkably, patients receiving TRF-budesonide experienced a reduction in the decline of eGFR after 9 months of therapy, along with a decrease in proteinuria after 24 months, compared to placebo [112].

#### 5.3.2. B-Cell Modulation

B cells are a target of high interest, due to their involvement in the production of anti-bodies. Similarly, BAFF and APRIL, both of them proteins related to the development and activation of B lymphocytes, are also interesting targets. Isolated BAFF inhibition, or dual inhibition of both BAFF and APRIL, may be effective targets for blocking autoantibody formation associated with the B-cell response, such as aberrant antibodies against Gd-IgA [113].

##### B-Cell-Directed Agents

Different phase I, II and III clinical trials assessing the efficacy and safety of APRIL antagonists in IgA nephropathy have recently been concluded, showing encouraging results in treating adult patients with IgA nephropathy (NCT03719443 and NCT03945318). Additionally, two dual inhibitors of BAFF and APRIL, atacicept and telitacicept, are currently undergoing evaluation in phase II studies in patients with IgA nephritis. However, preclinical studies are required to understand the role of APRIL and BAFF in IgAV before they can be evaluated as therapeutic targets [13,111] (Table 2).

Belimumab is a BLyS-neutralizing monoclonal antibody approved for the treatment of SLE and for preventing lupus nephritis that might have a potential role in this condition, and which is awaiting evaluation in clinical trials [111].

##### B-Cell-Depleting Agents

In this section, we must discuss two new agents: bortezomib and felzartamab. Bortezomib (BTZ) is a proteasome inhibitor approved for its use in the treatment of multiple myeloma. In a murine model of lupus-like disease, BTZ demonstrated reduction of proteinuria, decreased autoantibody production and increased survival [114]. A case report described an adult patient with refractory IgAV nephropathy successfully treated with BTZ [115]. Felzartamab is an anti-CD38 monoclonal antibody currently being evaluated in early-phase studies for IgA nephropathy, but data on IgAV are not available yet (Table 2).

#### 5.3.3. RAAS Inhibitors

RAAS plays a key role in cardiocirculatory and renal regulation. The long-term renoprotective effect of ACE inhibitors (ACEI) and ARB is well known, and they are used in patients with persistent proteinuria to reduce secondary glomerular damage [111,116]. A recent study in a pediatric population has shown higher levels of urinary aldosterone and angiotensin in IgAV than in healthy controls, with a significant increase in urinary angiotensinogen in patients with IgAV nephritis compared to those without, suggesting that the RAAS system may be triggered in IgAV, regardless of nephritis [111]. Overall, current evidence points to RAAS inhibition as a potential treatment line in IgAVN [111].

#### 5.3.4. Sodium–Glucose Cotransporter-2 Inhibitors (SGLT2-inhs)

SGLT2 inhibitors decrease the reabsorption of sodium and glucose via SGLT2 channels in the proximal tubule. This increases the delivery of sodium, chloride, and water to the macula densa, leading to tubule glomerular feedback. This decreases intra-glomerular perfusion pressure and overall filtration, contributing to a decline in urinary protein excretion. The renal protection offered by SGLT2 inhibitors is greater than that of RAAS blockers and is maintained in patients already treated with these drugs, regardless of whether or not they have type 2 diabetes [13].

In a recent study conducted by Dong et al. [117], 93 Chinese subjects with biopsy-proven IgA nephritis and persistent proteinuria underwent full supportive therapy, including optimal blood pressure control and a full dose of ACEI or ARB therapy. After three and six months of SGLT2-inh treatment, proteinuria decreased by 22.9% and 27.1%, respectively, relative to baseline [117]. However, to date, there are no data available on the use of SGLT2 inhibitors in IgAVN in children or adults.

#### 5.3.5. Endothelin Receptor Antagonists

Endothelins (ETs) are potent endogenous pressor agents comprising a family of three peptides, each one consisting of 21 amino acids: endothelin-1 (ET1), endothelin-2 (ET2), and endothelin-3 (ET3). ET1 is a powerful vasoconstrictor, synthesized by the vascular endothelium in response to several factors, whose actions are mediated by two receptors: ETA and ETB [118]. ET1 has proliferative effects on vascular smooth muscle cells (VSMCs), promotes fibroblast production, modulates extracellular matrix synthesis, induces VSMC hypertrophy, affects vascular permeability, mediates inflammation, and stimulates the sympathetic nervous system [118]. ET1 activates the ETA receptor across several kidney cell types, leading to vasoconstriction, podocyte injury, inflammation, and fibrosis, contributing to the progression of CKD [13]. All this is potentially involved in the pathogenesis of IgA nephropathy and, possibly, IgAVN.

There are three ETA/ETB receptor antagonists under study in glomerular diseases: sparsentan, atrasentan, and bosentan (Table 2). Sparsentan is a dual endothelin- and angiotensin-receptor antagonist with high selectivity for the ETA receptor and angiotensin II receptor type 1 [119]. In a double-blind, randomized, active-controlled phase 3 trial (PROTECT study), patients with IgA nephropathy were randomly assigned to receive 400 mg of oral sparsentan or 300 mg of oral irbesartan once daily. Interestingly, proteinuria was 40% lower in the sparsentan group compared to the irbesartan group after 110 weeks of therapy [119] (Table 2).

Atrasentan is another potent and selective ETA antagonist that has been shown to reduce proteinuria and preserve kidney function in patients with IgA nephritis at high risk of progression, whose results have not been published yet.

#### 5.3.6. Complement Pathway Inhibition

Emerging evidence supports the participation of the complement system in IgAVN, primarily through the lectin and alternative pathways [11,120]. In one study, 90% of cases exhibited IgA and C3 deposits within the glomeruli of the kidneys, together with a significant increase in C5 concentrations [121,122]. Additionally, amplified cleavage of C3 and C5 proteins was noted, correlating with elevated plasma concentrations of C3a and C5a in patients with IgAV [123].

Although clinical trials are currently underway to evaluate selective complement inhibitors such as avacopan, iptacopan, and narsoplimab in adults with IgA nephropathy with promising results, these agents have not been proved in patients with IgAV, yet. There is, however, one case report regarding the use of narsoplimab in a young woman with IgAVN-associated RPGN resulting in persistent reduction in lectin pathway activation and delayed deterioration of kidney function [124] (Table 2).

Avacopan, a selective C5a receptor antagonist, has demonstrated benefits in the treatment of ANCA-associated vasculitis. A pilot open-label phase II trial of avacopan in IgA nephritis patients showed improvement in proteinuria in 6 out of 7 patients during the treatment period [125]. However, further trials are needed to confirm the efficacy and safety of C5a inhibition in both IgA nephropathy and IgAVN (Table 2).

#### 5.3.7. Miscellaneous

##### Tonsillectomy

The tonsils play a significant role in regulating mucosal immunity following antigen exposure by stimulating polymeric IgA production. In vulnerable subjects with IgAV, this process may lead to increased production of Gd-IgA [11]. Consequently, it has been hypothesized that tonsillectomy could improve patient outcomes by alleviating abdominal and skin symptoms while reducing the urine protein-to-creatinine ratio (UPCR) in those with IgAV [126]. However, due to its invasive nature and the lack of convincing evidence regarding its efficacy in treating or preventing IgAV flares and related kidney complications, tonsillectomy is not currently recommended for managing this condition.

##### Gut Microbiota Modification

An important correlation exists between intestinal microbiota and mucosal immunity, particularly in patients with IgAV. Evidence indicates that the balance and composition of intestinal microbes are disturbed in these patients [127]. For instance, children with IgAV exhibiting different organ involvement demonstrate distinct patterns of intestinal microbiota. In a study by Li et al., children with joint or intestinal symptoms had increased levels of Proteobacteria and decreased levels of Actinobacteria compared to those with only skin symptoms [127].

Currently, there are no clinical trials assessing the potential role of modifying the gut microbiome in IgAV patients. As far as we know, there is only one ongoing clinical study investigating the role of fecal microbiota transplantation in adults with IgA nephropathy (NCT05182775), whose results have not yet been published. Additionally, a study exploring the effects of rifaximin, a non-absorbable antibiotic known to upregulate beneficial gut microbiota, has been conducted in a murine model of IgA nephropathy. Mice administered rifaximin showed decreased UPCR, reduced glomerular IgA1 deposition, and lower gut levels of BAFF and TNF-α mRNA expression [128].

## 6. Controversies and Conclusions

The management of patients with IgAV presents several controversies, particularly regarding treatment options and protocols. A relevant key area of debate includes the use of glucocorticoid therapy. In this regard, while glucocorticoids are commonly used as first-line therapy, their long-term use can lead to significant side effects. There is ongoing debate regarding the appropriate duration and dosage of glucocorticoids, particularly in cases with mild versus severe symptoms.

Another important issue is related to the role of immunosuppressive therapy. In this regard, the effectiveness and safety of immunomodulatory agents, such as mycophenolate mofetil, tacrolimus, and cyclosporine A, as glucocorticoid-sparing agents, is still debated. Clinicians often weigh the risks of immunosuppressants against the potential benefits in reducing glucocorticoid dependence. While leflunomide has shown promising results, there is still insufficient evidence on its long-term effectiveness and safety in the treatment of IgAV, leading to variability in clinical practice.

The use of biologic drugs, especially rituximab, has been shown to be effective in some studies in reducing relapse rates and achieving remission in both children and adults. However, their high cost and potential for adverse effects raise questions about their routine use and the selection criteria for patients who would benefit most.

There is general agreement among experts on the need for an individualized treatment approach: a personalized treatment plan is necessary, based on disease severity, patient characteristics, and potential comorbidities. The lack of standardized treatment protocols contributes to different practices among healthcare providers.

The potential of new emerging therapies, such as TRF-budesonide and sodium–glucose cotransporter-2 inhibitors, raises questions about their comparative efficacy and safety profiles, requiring further research to clarify their roles in therapy of patients with IgAV.

A particularly controversial issue is the management of renal involvement in IgAV. Decisions about initiation of dialysis, use of plasma exchange, and timing of immunosuppressive therapy can vary widely among clinicians.

Finally, a point to highlight is that the long-term outcomes of various therapies for IgAV remain uncertain, especially with regard to the risk of end-stage renal disease and other chronic complications.

## Figures and Tables

**Table 1:** Classification criteria for IgA vasculitis ^¶^.

ACR Classification Criteria * (Mills et al., 1990) Ref. [38]	EULAR/PRINTO/PRES Classification Criteria * (Ozen et al., 2010) Ref. [39]
**Two or more of the following**: - age ≤ 20 years ^1^ - palpable purpura (not related to thrombocytopenia) ^2^ - acute abdominal pain: generally diffuse and worsens with Meals ^3^ - skin biopsy showing granulocytes inside small arteriolar or venular walls Sensitivity 87.1%; specificity 87.7%	**MANDATORY**: Purpura or petechiae with lower limb predominance ^2^ AND **One or more of the following 4 criteria**: - abdominal pain (diffuse with acute onset) ^3^ - arthritis or arthralgia (of acute onset) ^4^ - kidney involvement (proteinuria and/or hematuria) ^5^ - leucocytoclastic vasculitis with predominant IgA deposits or proliferative GMN with predominant IgA deposits Sensitivity 100%; specificity 87%

^1^ Onset of the first symptoms at the age of 20 or less. ^2^ Usually located on the buttocks, legs, or feet, but may also appear in other areas. ^3^ Often crampy in nature and may be associated with gastrointestinal manifestations such as nausea or vomiting. ^4^ Joint pain (arthralgia) or swelling (arthritis), commonly affecting the knees and ankles. ^5^ May include hematuria, proteinuria, or renal failure, as evidenced by laboratory tests. * The criteria were used primarily for children, but may also apply to adults. ^¶^ This table has been modified from Hočevar et al. ref. [40]. GMN: glomerulonephritis.

**Table 2:** Potential future drugs for IgA vasculitis treatment.

Regulation of mucosal immunity - Tonsillectomy - Gut microbiota modification - Targeted-release formulation (TRF) of budesonide
B-cell modulation - B-cell-directed therapy - BAFF-neutralizing monoclonal antibodies - APRIL-neutralizing monoclonal antibodies: sibeprenlimab; zigakibart - Dual antagonists of BAFF and APRIL: atacicept; telitacicept; povetacicept - BLyS-neutralizing monoclonal antibody: belimumab - B-cell-depleting agents - Humanized anti-CD20 monoclonal antibody: ofatumumab - Bortezomib (proteasome inhibitor); felzartamab (anti-CD38 monoclonal antibody)
SYK inhibition - Fostamanitib
T-cell modulation
RAAS inhibitors - Angiotensin-converting enzyme inhibitors (ACEI) - Angiotensin receptor blockers (ARB) - New ACEI: benazepril
Sodium-Glucose Cotransporter-2 Inhibitors (SGLT2-inh) - Dapaglifozin and empaglifozin
Endothelin receptor antagonists - Spasertan; atrasentan; bosentan
Complement pathways inhibitors - Lectin pathway inhibition: narsoplimab (MASP-2 inhibition) - Alternative pathway inhibition: iptacopan; vermicopan - Terminal pathway inhibition: pegcetacoplan; ravulizumab; cemdisiran; avacopan
Unclassified agents - Mizoribine (reduces DNA synthesis and decreases the proliferation of B and T cells)

Abbreviations: ACEI: angiotensin-converting enzyme inhibitors; APRIL: a proliferation-inducing ligand; ARB: Angiotensin receptor blockers; BAFF: B-cell activating factor; BLyS: B-lymphocyte stimulator; DNA: deoxyribonucleic acid; IgA: immunoglobulin A; MASP-2: MBL (mannose-binding lectin)-associated serine protease 2; RAAS: renin-angiotensin-aldosterone system; SGLT2-inh: Sodium-Glucose Cotransporter-2 Inhibitors; SYK: spleen tyrosine kinase; TRF: Targeted-release formulation.

## Data Availability

No new data were created or analyzed in this study.
